# Accurate and robust segmentation of neuroanatomy in T1‐weighted MRI by combining spatial priors with deep convolutional neural networks

**DOI:** 10.1002/hbm.24803

**Published:** 2019-10-21

**Authors:** Philip Novosad, Vladimir Fonov, D. Louis Collins, Alzheimer's Disease Neuroimaging Initiative

**Affiliations:** ^1^ McConnell Brain Imaging Centre Montreal Neurological Institute, McGill University Montreal Canada; ^2^ Department of Biomedical Engineering McGill University Montreal Canada

**Keywords:** deep learning, magnetic resonance imaging, neural networks, neuroanatomy, segmentation, spatial priors

## Abstract

Neuroanatomical segmentation in magnetic resonance imaging (MRI) of the brain is a prerequisite for quantitative volume, thickness, and shape measurements, as well as an important intermediate step in many preprocessing pipelines. This work introduces a new highly accurate and versatile method based on 3D convolutional neural networks for the automatic segmentation of neuroanatomy in T1‐weighted MRI. In combination with a deep 3D fully convolutional architecture, efficient linear registration‐derived spatial priors are used to incorporate additional spatial context into the network. An aggressive data augmentation scheme using random elastic deformations is also used to regularize the networks, allowing for excellent performance even in cases where only limited labeled training data are available. Applied to hippocampus segmentation in an elderly population (mean Dice coefficient = 92.1%) and subcortical segmentation in a healthy adult population (mean Dice coefficient = 89.5%), we demonstrate new state‐of‐the‐art accuracies and a high robustness to outliers. Further validation on a multistructure segmentation task in a scan–rescan dataset demonstrates accuracy (mean Dice coefficient = 86.6%) similar to the scan–rescan reliability of expert manual segmentations (mean Dice coefficient = 86.9%), and improved reliability compared to both expert manual segmentations and automated segmentations using FIRST. Furthermore, our method maintains a highly competitive runtime performance (e.g., requiring only 10 s for left/right hippocampal segmentation in 1 × 1 × 1 mm^3^ MNI stereotaxic space), orders of magnitude faster than conventional multiatlas segmentation methods.

## 1 INTRODUCTION

Accurate structural segmentation of magnetic resonance (MR) brain images is essential for volume, thickness, and shape measurements. Such quantitative measurements are widely used in neuroscience to characterize structural changes associated with age and disease. Given the often prohibitive cost of consistent and reliable expert manual segmentations, a vast number of diverse and fully automated segmentation methods have been proposed. While earlier segmentation methods generally employed various heuristics tailored for the segmentation task at hand, more recent segmentation methods are more accurate and attempt to transfer labels from a set of expertly labeled images (atlases) to the target image. Some such methods have attempted to learn complex mappings between image features and labels using traditional machine‐learning based classifiers (e.g., support vector machines (Boser, Guyon, & Vapnik, 1992) and random forests (Breiman, 2001)) combined with handcrafted feature sets (Morra et al., 2010; Zikic et al., 2012), while others have found success transferring labels using a combination of linear or nonlinear image registration with local and/or nonlocal label fusion (so‐called “multiatlas segmentation” methods (Coupé et al., 2011, Heckemann, Hajnal, Aljabar, Rueckert, & Hammers, 2006, Iglesias & Sabuncu, 2015)). Indeed, many state‐of‐the‐art results (e.g., hippocampus segmentation (Zandifar, Fonov, Coupé, Pruessner, & Collins, 2017) and brain extraction (Novosad & Collins, 2018) exploit a complementary combination of both multiatlas segmentation and machine‐learning methods (e.g., error correction (EC) (Wang et al., 2011)).

More recently, convolutional neural networks (CNNs) (LeCun et al., 1989) have been used for MR image segmentation, obtaining similar or better performance compared to the previous state of the art while requiring only a fraction of the processing time (despite long training times). CNNs are particularly attractive because they have the potential to model more complicated functions without the need for handcrafted feature sets, instead autonomously learning to extract task‐dependent discriminative features from the training data. Also in contrast to traditional machine‐learning classifiers, by stacking many convolutional layers sequentially and/or by incorporating downsampling operations into the network architectures, CNNs have the capacity to model increasingly complex and long‐range spatial relationships in the input, contributing to their excellent performance on image segmentation tasks in particular. However, repeated convolutions and/or downsampling steps produce coarse features, leading to low‐resolution segmentations that can be particularly problematic when targeting smaller structures. Therefore, explicitly multiscale architectures are often preferred, which are capable of preserving local detail while still enabling the modeling of complex long‐range spatial relationships. For example, Kamnitsas et al. (2017) and Ghafoorian et al. (2017) both adopt multiscale, multipath architectures which take as input patches extracted at different resolutions, and perform late fusion between the extracted features from the different resolutions. Other works adapt popular architectures such as the U‐Net (Ronneberger, Fischer, & Brox, 2015) (adapted in Guha Roy, Conjeti, Navab, and Wachinger (2018)) and DenseNet (Huang, Liu, van der Maaten, & Weinberger, 2017) (adapted in Dolz, Ayed, Yuan et al. (2017), Dolz, Desrosiers, and Ben Ayed (2017), and Dolz, Desrosiers, Wang et al. (2017)), both of which use skip connections in order to leverage multiscale information.

Due to hardware limitations of modern graphics processing units (GPUs), modern volumetric medical images (e.g., MR or tomography scans) typically cannot fit into memory, and need to be subsampled in order to be processed by a CNN. Most commonly, 3D networks are trained on smaller 3D patches (Dolz, Desrosiers, & Ben Ayed, 2017; Kamnitsas et al., 2017), or 2D networks on single 2D slices (Guha Roy et al., 2018). Therefore, despite the development of recent architectures which are capable of better modeling complex, long‐range and multiscale spatial relationships in the input, the implicit spatial context available to the network is still limited, and it is often useful to explicitly provide the network with additional spatial contextual information. Examples of applications which leverage spatial contextual features include that of de Brebisson and Montana (2015), which incorporates distances from pre‐defined neuroanatomical structures, that of Wachinger, Reuter, and Klein (2017), which incorporates spatial and spectral coordinates (by computing eigenfunctions of the Laplace–Beltrami operator on a pre‐estimated brain mask), that of Kushibar et al. (2018), which incorporates nonlinear‐registration‐based atlas probabilities, and that of Ghafoorian, Karssemeijer, et al. (2017), which incorporates a handcrafted combination of such features. While the addition of such features has been shown to result in better performance, the computation of such features is often extremely expensive relative to the time required to apply a trained CNN, limiting the efficiency of the methods.

In this work, we propose a novel CNN‐based method for the automated segmentation of neuroanatomy in brain MR images. To maximize spatial context available to the network, we combine a deep 3D fully CNN with dense connections for multiscale processing with explicitly provided spatial contextual information through the use of linear‐registration‐derived spatial priors. We furthermore regularize our trained networks with a data augmentation scheme based on random elastic deformations, increasing the generalizability of the trained networks particularly in cases where limited labeled training subjects are available. We extensively validate our method on three neuroanatomical segmentation tasks using different manually labeled datasets, showing in each case consistently more accurate and robust performance compared to state‐of‐the‐art multiatlas segmentation and other CNN‐based methods, while maintaining a highly competitive runtime performance. Using a scan–rescan dataset, we also demonstrate that our proposed method achieves excellent scan–rescan reliability, with an accuracy comparable to the scan–rescan reliability of repeated expert manual segmentations.

## 2 METHODS AND MATERIALS

### 2.1 Baseline network

In contrast to traditional machine‐learning classifiers which treat their inputs as unordered vectors, CNNs explicitly treat their inputs as spatially structured images and work by extracting hierarchical and discriminative representations using sequential applications of the core building‐block known as a “convolutional layer” (LeCun et al., 1989). The function of a convolutional layer is to convolve its input with multiple learned filters and then apply a nonlinear activation function (otherwise, the network would just learn a linear transform of the input data). Assuming a simplified network architecture consisting only of convolutional layers, the convolutional filter $W_{l}^{k,n}$ at network layer *l* is applied across all the *m*_*l*−1_ feature maps produced by the previous convolutional layer *l*−1, resulting in a new set of feature maps, to which a position‐wise nonlinear activation function *f*() is applied. For example, *k*th output feature map at layer *l* is given by:

(1) $$y_{l}^{k}=f\left(\sum_{n=1}^{m_{l-1}}W_{l}^{k,n}*x_{l-1}^{n}+b_{l}^{k}\right)$$

where *m*_*l*_ is the number of convolutional filters in layer *l*, $x_{l-1}^{n}$ is the *n*th feature map of the input to layer *l*, $W_{l}^{k,n}$ is the *k*th learnable filter, and $b_{l}^{k}$ is the learnable bias.

We take as our starting point a 3D fully convolutional network, variants of which have shown success in tasks such as brain tumor and ischemic stroke lesion segmentation (Kamnitsas et al., 2017), as well as subcortical structure segmentation (Dolz, Desrosiers, & Ben Ayed, 2017). Instead of using fully connected layers and predicting the label of only one or several voxels for each input patch (Ghafoorian, Karssemeijer, et al., 2017; Kushibar et al., 2018; Wachinger et al., 2017), fully convolutional networks discard fully connected layers and produce dense label estimates for whole patches at a time. Consequently, fully convolutional networks have many fewer parameters (and are therefore less prone to overfitting) and preserve the spatial structure of the input. Also following Kamnitsas et al. (2017) and Dolz, Desrosiers, and Ben Ayed (2017), we entirely avoid downsampling or max‐pooling layers to preserve the spatial resolution of the output segmentations.

**Figure 1:**
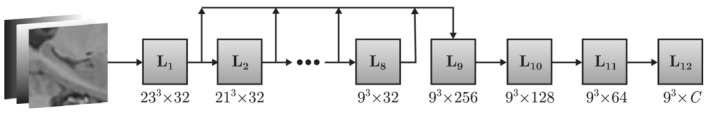
Schematic of baseline architecture. The network takes as input a 25^3^ × *N* patch (here, spatial coordinates patches are concatenated with the input image patch as described in Section 2.2.1) and returns a multichannel probabilistic label estimate for the central 9^3^ voxels. The dimensionality of the output of each layer is reported as *size* × *number of feature maps*. Detailed specifications for each layer are reported in Table 1

Our baseline architecture takes as input a 3D patch with size 25^3^ and *N* channels (e.g., different MR imaging (MRI) contrasts or priors [Section 2.2.1]), and returns a smaller 3D patch label estimate with volume 9^3^ × *C* centered on the same respective spatial coordinates in image space, where *C* is the number of classes. Figure 1 depicts the network architecture schematically, and detailed architectural specifications (including the number of filters and the activation function at each convolutional layer) are provided in Table 1. First, a series of convolutional layers (*L*_1_ through *L*_8_ in Figure 1 and Table 1) with filters of size 3 × 3 × 3 are applied, without padding and with unit stride (in order to preserve spatial resolution). We note that therefore each application of a 3 × 3 × 3 convolution layer reduces the size of resultant input feature maps by two voxels in each dimension: after eight 3 × 3 × 3 convolutional layers, the size of the feature maps is therefore reduced from 25^3^ to 9^3^. While padding could be used prior to each convolution in order to preserve the size of the feature maps throughout the network, like the work of Kamnitsas et al. (2017) and Dolz, Desrosiers, and Ben Ayed (2017), we opted against this approach, allowing us to explore deeper architectures that would otherwise be prohibitively expensive in terms of GPU memory.

**Table 1:** Baseline network architecture specifications. The network has roughly 220,000 parameters (depending on the number of classes *C* and the number of input channels *N*). For each layer, if applicable, the size and number of learnable filters is reported in the third column as (*filter size*) × *number of filters*. A corresponding schematic depiction of the network architecture is shown in Figure 1

	Operation	Filters	Nonlinearity	Input dimension	Output dimension	Notes
*L*_1_	Convolution	(3 × 3 × 3) × 32	ELU	25 × 25 × 25 × *N*	23 × 23 × 23 × 32	—
L_2_–*L*_8_	Convolution	(3 × 3 × 3) × 32	ELU	23 × 23 × 23 × 32	9 × 9 × 9 × 32	—
*L*_9_	Dense connection	—	—	(9 × 9 × 9 × 32) × 8	9 × 9 × 9 × 256	BN
*L*_10_	Convolution	(1 × 1 × 1) × 128	ELU	9 × 9 × 9 × 256	9 × 9 × 9 × 128	DO
*L*_11_	Convolution	(1 × 1 × 1) × 64	ELU	9 × 9 × 9 × 128	9 × 9 × 9 × 64	DO
*L*_12_	Convolution	(1 × 1 × 1) × *C*	Spatial softmax	9 × 9 × 9 × 64	9 × 9 × 9 × *C*	—

Abbreviations: BN, batch normalization; DO, dropout (with dropout probability 0.1); ELU, exponential linear unit.

While the first layers of a CNN extract high‐resolution feature maps which respond to basic local image features such as edges, due to repeated convolution, the feature maps extracted from the deeper layers tend to have lower resolution and respond to more global and abstract image features. Ideally, a classifier should consider features extracted across all scales of the input, that is, features extracted from each convolutional layer rather than only the last. To this end, similar to Dolz, Desrosiers, and Ben Ayed (2017), we follow Huang et al. (2017) and use a “dense connection” after layer *L*_8_ which consists of the channel‐wise concatenation of the feature maps produced by the preceding convolutional layers *L*_1_ through *L*_8_. In this way, the last convolutional layers following the dense connection have direct access to the multiscale feature maps produced by each the preceding convolutional layers, and are therefore capable of maintaining feature maps with high spatial resolution while also considering complex and long‐range characteristics of the input. The dense connection also encourages feature reuse and improves the convergence properties of the network during training by providing a more direct path during backpropagation between the calculated loss and the earlier convolutional layers (Huang et al., 2017). We note that since the output feature maps produced by each convolutional layers have different sizes, only the central 9^3^ voxels of each feature map are concatenated. The concatenated feature maps are then batch normalized (Ioffe & Szegedy, 2015) to make the first and second order statistics of feature maps consistent across channels and layers, improving convergence.

The batch‐normalized concatenated feature maps are then further processed by two convolutional layers (*L*_10_ and *L*_11_ in Figure 1 and Table 1) with filters of size 1 × 1 × 1. These layers serve to model inter‐channel (and therefore also multiscale) dependencies and also to reduce the number of feature maps prior to being fed into final classification layer. Dropout (Srivastava, Hinton, Krizhevsky, Sutskever, & Salakhutdinov, 2014) (with drop probability *p* = 0.1) is applied after both *L*_10_ and *L*_11_ to help regularize the model. The final classification layer (*L*_12_ in Figure 1 and Table 1) processes the resulting features using a set of *C* filters (where *C* is the number of classes under consideration) of size 1 × 1 × 1, producing a probabilistic label estimate image *p*_*c*_ of size 9 × 9 × 9 for each class *c*.

Network parameters (i.e., convolutional filters and biases) are estimated iteratively by optimizing the loss function in Equation (2) using a gradient‐descent optimizer over minibatches of size *B*. The loss function ℒ to be minimized is defined as:

(2) $$ℒ=J+\alpha {\left\|W\right\|}_{2}^{2}$$

where *α* (empirically set to 1 × 10^−4^ in our experiments) penalizes the *l*_2_ norm of the network filters *W*, reducing overfitting, and *J* is the categorical cross‐entropy loss:

(3) $$J=-\frac{1}{B\times V}\sum_{c=1}^{C}\sum_{b=1}^{B}\sum_{v=1}^{V}c_{b}^{v}\log p_{c_{b}^{v}}$$

where $p_{c_{b}^{v}}$ is the output of the final classification layer for voxel *v* and class *c*, and $c_{b}^{v}$ is the corresponding reference label. The training procedure is further detailed in Section 2.4.

### 2.2 Adding spatial priors

#### 2.2.1 Spatial coordinates

Though other works have explored the effects of augmenting their architectures with spatial coordinates and/or spatial probability maps, this is typically accomplished by concatenating a single vector to the output of a flattened fully connected layer (Ghafoorian, Karssemeijer, et al., 2017; Kushibar et al., 2018; Wachinger et al., 2017) which has no analogue in the proposed network. Furthermore, as our fully convolutional network makes predictions for whole patches rather than one or several voxels, the features associated with each voxel should be augmented with their respective spatial coordinates rather than that of the central voxel only. We therefore make use of whole spatial coordinate patches: given an input patch centered on spatial coordinate (*x*,*y*,*z*) in image space, we extract three additional patches (with the same spatial dimensions as the input patch) centered on spatial coordinate (*x*,*y*,*z*) from each of three “coordinate images.” For example, for the *x*‐coordinate image, the value at spatial coordinate (*x*,*y*,*z*) is simply *x*, and similar for the *y*‐ and *z*‐coordinate images. The three spatial coordinate patches are then concatenated with the image intensity patch, forming a multichannel input (e.g., *N* = 4 channels for a single MRI modality with spatial coordinate priors) before being fed into the first layer of the network.

#### 2.2.2 Working volumes

In order to benefit from the explicit incorporation of spatial coordinates into the network input, it is important that all images are registered to a common space. For anatomically regular structures which present relatively little variation in shape and location after registration, we can further take advantage of this spatial alignment by defining, given a set of training subjects, a working volume in which patches are extracted when training and applying the networks.

For each structure of interest *c*, we first obtain a class‐specific boundary‐like working volume *B* ^*c*^ by subtracting the union $U^{c}=\cup_{i=1}^{I}M_{i}^{c}$ from the intersection $I^{c}=\cap_{i=1}^{I}M_{i}^{c}$ of all training subject labels $M_{i}^{c}$ for the given structure of interest. In other words, each class‐specific working volume includes only voxels for which there is inconsistency among the training subject labels with respect to the presence of *c*, and therefore highlights the spatial region where the absence or presence of the respective structure is uncertain. In our experiments, we opt to conservatively dilate each working volume to ensure that it is appropriate for (unseen) test subjects, that is, *B* ^*c*^ = *D* ⊕ (*U* ^*c*^−*I* ^*c*^), where *D* is the dilation structuring element (here set to 3 × 3 × 3 voxels). Finally, the spatial region in which the presence of the respective structure is certain is further stored as a positive volume *P* ^*c*^ = *I* ^*c*^ −(*I* ^*c*^ ⋂ *B* ^*c*^), for use at test time.

**Figure 2:**
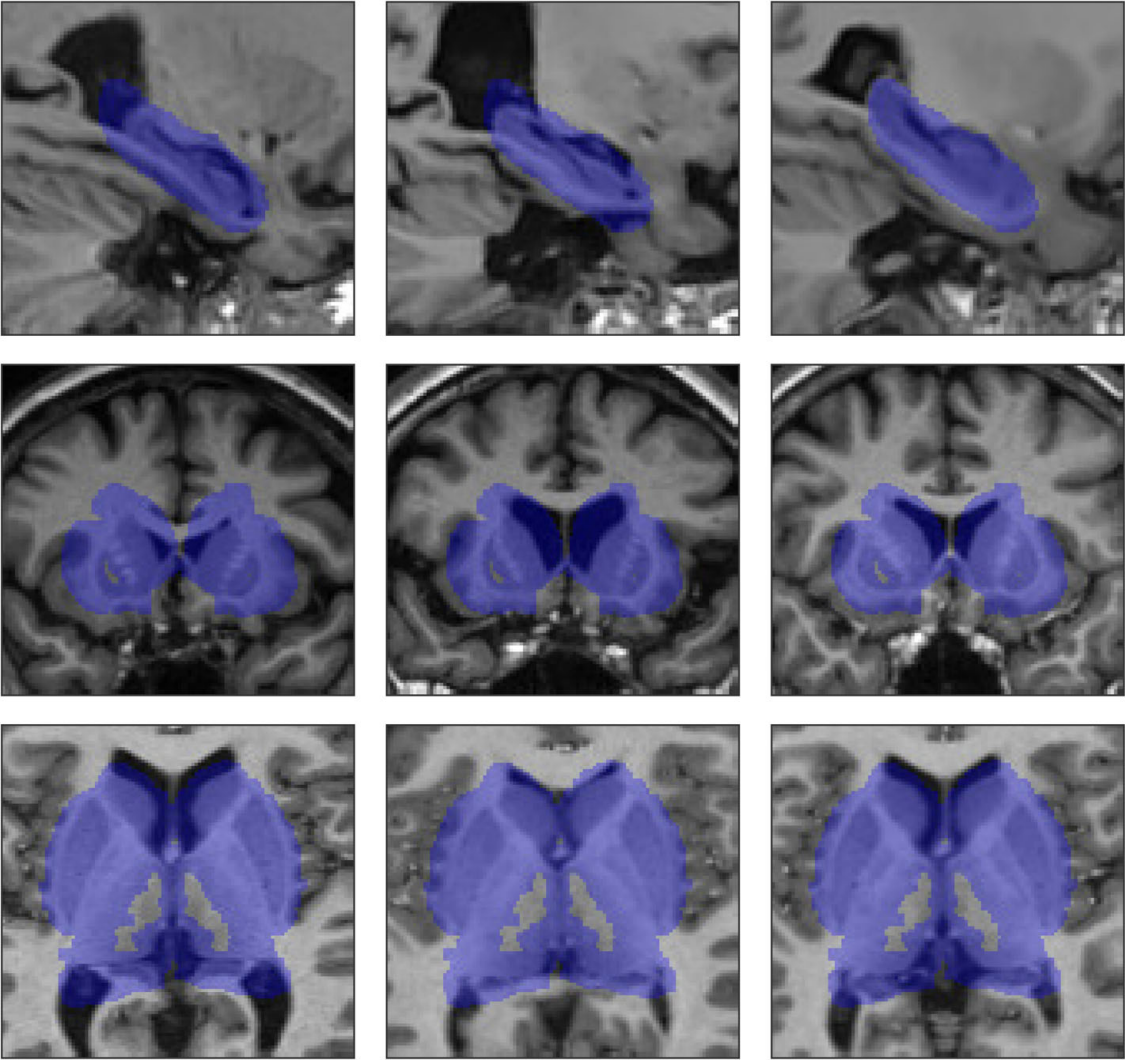
Example working volumes overlaid on random subjects from the hippocampus (top row), subcortical (middle row), and multistructure (bottom row) segmentation experiments [Color figure can be viewed at http://wileyonlinelibrary.com]

A final working volume *B* is obtained by forming the union of all class‐specific working volumes, that is, $B=\cup_{c=1}^{C}B^{c}$. Example working volumes are shown in Figure 2. Using working volumes has two primary advantages. First, during training, all samples are drawn from the working volume *B* (such samples are approximately class‐balanced, as described in Section 2.4), forcing the network to learn from more challenging examples. Second, at test time, the network is only required to evaluate the uncertain voxels contained within the working volume, decreasing processing time. We note that to obtain the final label estimate for a test subject, the label estimate within the working volume must be combined with the positive volumes (e.g., the inner core of the thalami in the third row of Figure 2).

### 2.3 Data augmentation with random elastic deformations

Deep neural networks, which have a high modeling capacity, are particularly dependent on the availability of large quantities of labeled training data in order to generalize well to new unseen test data. In the context of MRI segmentation, low numbers of training samples are typically encountered due to the high cost of generating manually annotated data. To remedy this problem, data augmentation can be used to artificially expand the training set. Commonly, this is accomplished by applying user‐specified but label‐preserving transformations to the training data, such as reflections, rotations, and flips. However, since in the present work, all images are linearly registered in a common space and are therefore approximately the same size and with the same orientation, these transformations would be counterproductive. Rather, the relevant differences between linearly registered images are local and nonlinear in nature. To create plausible synthetic training samples, we therefore chose to apply random 3D elastic deformations using a method based on Simard, Steinkraus, and Platt (2003).

To generate a random elastic deformation, we first generate a 3D vector field (where each vector element specifies the per‐pixel displacement in each of the *x*, *y*, and *z* directions, respectively) with the same spatial dimensions as the input samples, and then assign each vector element a random value selected from the uniform distribution *U*(−1,1). The vector field is then smoothed in each direction using Gaussian kernels with *standard deviation σ*_*e*_ (controlling the elasticity of the deformation), normalized to have a mean per‐pixel displacement of one, and then multiplied by a constant *α*_*i*_ (controlling the intensity of the deformation), producing the final deformation. During training, we apply data augmentation on the fly by generating a different random data augmentation transformation for each sample prior to being fed through the network. The random elastic deformation is then used to interpolate each training sample (i.e., the image appearance patch, the three spatial coordinates patches, and the reference label image) using linear interpolation. We note that applying linear interpolation introduces a slight blurring in the label images, which itself can be useful as a regularization technique (Szegedy, Vanhoucke, Ioffe, Shlens, & Wojna, 2016). The parameters *σ*_*e*_ and *α*_*i*_ were determined using a coarse grid search, detailed in Section 3.1.3.

### 2.4 Training and testing

Network parameters are optimized iteratively using RMSProp (Tieleman & Hinton, 2012), an adaptive stochastic gradient descent algorithm, with Nesterov momentum (Nesterov, 1983) (momentum = 0.9) for acceleration. At each training iteration, we sample approximately 2,000 voxels, with an equal number of voxels sampled from each training subject. Since CNNs are sensitive to class imbalance, we sample an equal number of voxels from each structure (background included). Training samples (i.e., whole patches) are then extracted around each selected voxel, and image appearance patches are individually normalized to zero mean and unit *SD*. All training samples are then randomly shuffled and processed by the network in batches of size *B*. Network weights are randomly initialized with the Glorot method (Glorot & Bengio, 2010), and all biases are initialized to zero. Training was performed on a single NVIDIA TITAN X with 12 GB GPU memory. Software was coded in Python, and used Lasagne (Dieleman et al., 2015), a lightweight library to build and train the neural networks in Theano (Al‐Rfou et al., 2016).

To counter overfitting, we employ the early stopping technique, whereby a randomly selected validation subject set (taken here to be 20%) is held out from the training subject set. Before training, a fixed validation set is obtained by randomly sampling a fixed number of patches from each validation subject within the working volume. Unlike during the training phase, we extract the validation set uniformly (i.e., without enforcing class balance), so that the distribution of classes in the validation set better approximates the true distribution of classes within the working volume. During training, at each iteration, the average categorical cross‐entropy loss (Equation (3)) over the validation set is measured. The final weights for the trained model are taken from the iteration which achieved the lowest validation loss, and training is stopped if the previously attained lowest validation error does not further decrease after 30 iterations. A static learning rate of 2.5 × 10^−4^ is used for training the networks. This value was empirically determined in our preliminary experiments following the suggestions described by Bengio (2012), i.e., by roughly finding the smallest learning rate which caused training to diverge, and then dividing it in half. When training the baseline network, we process the samples in smaller batches of size *B* = 128.

At testing, we apply the trained network with a stride of four voxels in each dimension, averaging the patch label estimates where they overlap. Further reducing the stride (i.e., increasing the overlap between patch label estimates) did not significantly improve performance in preliminary studies. We further apply a fast and simple postprocessing step which consists in only keeping the largest connected component for each label, thereby eliminating isolated clusters of false positives.

## 3 EXPERIMENTS AND RESULTS

We first assessed the impact of spatial priors, architecture depth and width, and data augmentation on the task of hippocampal segmentation in the ADNI‐1 (http://adni.loni.usc.edu) (Jack et al., 2008; Mueller et al., 2005) dataset. To demonstrate the versatility of the proposed segmentation method, we further applied it subcortical segmentation using the IBSR (http://www.nitrc.org/projects/ibsr/) dataset, and multistructure segmentation using the OASIS (https://www.oasis-brains.org/) (Marcus et al., 2007) scan–rescan dataset. Dataset and preprocessing specifications are provided in the respective sections below.

We assess segmentation accuracy and reliability using the Dice coefficient. The Dice coefficient measures the extent of spatial overlap between two binary images:

(4) $$\text{Dice}=100\%\times 2\left|A\cap R\right|/\left(\left|A\right|+\left|R\right|\right)$$

where *A* is an automatically segmented label image, *R* is the reference label image, ∩ is the intersection, and |·| counts the number of nonzero elements. We here express the Dice coefficient as a percentage, with 100% indicating perfect overlap. For multilabel images, we compute the Dice coefficient for each structure independently.

Segmentations with high general overlap may nonetheless have clinically important differences in their boundaries. To measure these differences, we also use the modified Hausdorff distance (MHD) (Dubuisson & Jain, 1994):

(5) $$\mathrm{MHD}=\max\left(h\left(A,R\right),h\left(R,A\right)\right)$$

where *h*(*A*,*R*) is the mean distance of the set of minimum distances between each labeled voxel in *A* and its nearest labeled voxel in *R*; *h*(*R*,*A*) is computed similarly. For the MHD, lower values are better.

Finally, we assess the statistical significance of differences between distributions of Dice coefficients and MHD values using nonparametric Wilcoxon signed‐rank tests.

### 3.1 Application to hippocampus segmentation: Effect of spatial priors, architecture, and data augmentation

The hippocampal dataset consists of 60 T1‐weighted (T1w) 1.5 T scans (acquired using an MP‐RAGE sequence [Mugler III & Brookeman, 1990]) from the multicenter ADNI‐1 dataset, each with manually segmented (Pruessner et al., 2000) left and right hippocampi. Twenty subjects were selected from each of the following clinical subgroups: normal controls, mild cognitive impairment, and Alzheimer's disease. Since this dataset was previously used to compare several state‐of‐the‐art algorithms (Zandifar et al., 2017), for our experiments, we use the same data (e.g., previously preprocessed as described in Zandifar et al.) to enable meaningful comparisons with the results reported in the aforementioned work. Pre‐processing consisted of patch‐based (PB) denoising (Coupé et al., 2008), N3 nonuniformity correction (Sled, Zijdenbos, & Evans, 1998), linear intensity normalization to the range [0,100], and affine registration to the MNI‐ICBM152 template (Fonov et al., 2011) with 1 × 1 × 1 mm^3^ resolution. We trained our networks to segment both the right and left hippocampi. To obtain a segmentation for each subject, we carried out a fivefold cross‐validation (i.e., 48 training subjects and 12 testing subjects per fold) with each fold containing the same number of subjects from each clinical subgroup.

#### 3.1.1 Effect of spatial priors

Mean Dice and MHD values for several variants of the baseline network (CNN‐B) are reported in Table 2. We also include in Table 2 results without the postprocessing step (keeping only the largest connected component for each segmented structure, i.e. removing isolated clusters of false positives). Postprocessing was crucial for obtaining good performance with CNN‐B, (reducing the mean MHD from 4.59 to 0.27 mm and increasing mean Dice from 87.2 to 90.6%, *p* < 1 × 10^−20^), but produced more subtle improvements when applied to the methods using either the working volumes or spatial coordinates, and smaller improvements still when applied to the method incorporating both spatial priors. Nonetheless, the effect of postprocessing remained statistically significant (*p* < 1 × 10^−4^) with respect to mean MHD and mean Dice when applied to the latter method. For the fairest possible comparisons, we use postprocessing in all CNN‐based methods for the subsequent experiments.

**Table 2:** Effect of augmenting the CNN‐B with CNN‐SC alone, the CNN‐WV alone, and both CNN‐SP on network performance for the hippocampus segmentation experiment

	Dice (%)	MHD (mm)
CNN‐B	90.2 (2.8)	0.27 (0.10)
CNN‐B^a^	87.2 (3.2)	4.59 (2.47)
CNN‐WV	90.9 (2.4)	0.26 (0.13)
CNN‐WV^a^	90.6 (2.5)	0.54 (0.59)
CNN‐SC	91.3 (2.1)	0.24 (0.09)
CNN‐SC^a^	90.1 (2.6)	0.64 (0.40)
CNN‐SP	91.5 (2.0)	0.23 (0.08)
CNN‐SP^a^	91.4 (2.1)	0.25 (0.14)

*Note*: Mean Dice and MHD values over both left and right hippocampi are reported, with *SD*s in parentheses.

Abbreviations: CNN, convolutional neural network; CNN‐B, baseline CNN; CNN‐SC, CNN with spatial coordinates; CNN‐SP, CNN with both spatial priors; CNN‐WV, CNN with working volume; MHD, modified Hausdorff distance.

a No postprocessing was performed.

**Figure 3:**
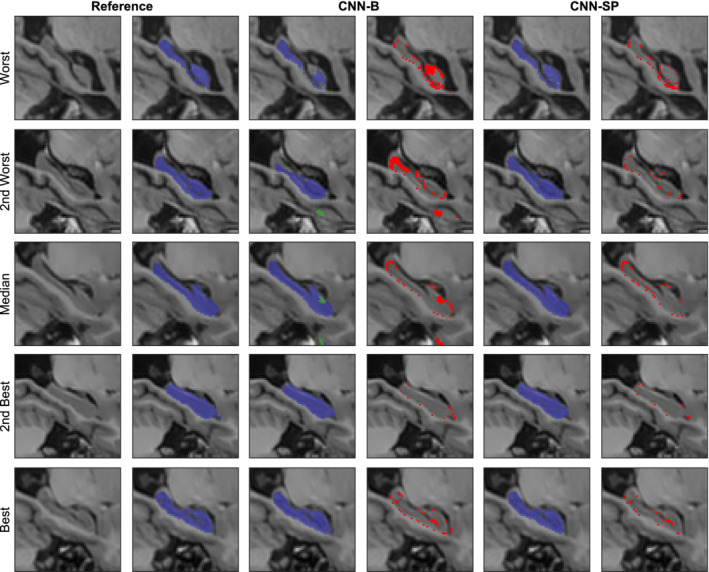
Example right hippocampus segmentations and respective errors using the baseline network (CNN‐B), and the network augmented with both the working volume and spatial coordinates (CNN‐SP). The subjects with the worst, second worst, median, second best, and best overlap after applying CNN‐B are shown for comparison. The left hippocampus is overlaid in green, and the right hippocampus in blue. Errors are overlaid in red in columns four and six [Color figure can be viewed at http://wileyonlinelibrary.com]

Augmenting CNN‐B with spatial coordinates (CNN‐SC) only or using working volumes (CNN‐WV) only both resulted in statistically significant (*p* ≤ 1 × 10^−3^) increases in performance with respect to both mean Dice and mean MHD. Combining both spatial priors (CNN‐SP) resulted in the best performance (mean Dice = 91.5%, mean MHD = 0.23 mm), a statistically significant improvement over both CNN‐SC and CNN‐WV with respect to both mean Dice (*p* ≤ 0.005) and mean MHD (*p* < 0.01). Using the working volume also significantly reduced the mean processing time from 28.3 ± 1.7 to 3.5 ± 0.5 s per subject. Example segmentations showing the improvements due to the use of spatial priors are displayed in Figure 3. These segmentations are shown without postprocessing (which consists of keeping only the largest connected component for each label) to better understand the nature of the errors made by the different methods. Without spatial priors, the baseline network CNN‐B had difficulty distinguishing between left and right hippocampi, producing isolated clusters of false positives, and, in the worst cases, mistook contiguous hippocampal gray matter for background. The addition of spatial priors largely avoided these errors, and helped to produce smoother and more anatomically regular segmentations.

#### 3.1.2 Effect of network architecture

We assessed whether the performance of the CNN‐SP method could be further improved by widening (learning more filters in the 3 × 3 × 3 convolutional layers) or deepening (including more 3 × 3 × 3 convolutional layers) the network architecture. For the deeper networks, we correspondingly increased the size of the input samples in order to preserve the output size, that is, for each additional 3 × 3 × 3 convolutional layer, the size of the input was increased by two voxels in each spatial dimension). Because of the increased memory requirements associated with deeper architectures, during training, we reduced the batch size to *B* = 32 as needed. Quantitative results are reported in Table 3. While widening the network by doubling the number of learnable filters (from 32 to 64) in convolutional layers *L*_1_ through *L*_8_ produced no appreciable gain in performance, deepening the network by increasing the number of 3 × 3 × 3 convolutional layers resulted in a gradual increase in performance with respect to both mean Dice and MHD, with a plateau reached when using fourteen or sixteen 3 × 3 × 3 convolutional layers (corresponding to input samples with spatial dimensions of 39^3^ and 41^3^ voxels, respectively). The mean processing time of the deepest network was correspondingly higher, requiring 10.4 ± 0.5 s per subject with twenty 3 × 3 × 3 convolutional layers, compared to CNN‐SP which required 3.5 ± 0.5 s per subject with only twelve 3 × 3 × 3 convolutional layers. For subsequent experiments, we opt to evaluate the deepest network, and for brevity denote the architecture by “CNN‐SP‐D.”

**Table 3:** Effect of widening and deepening the CNN‐SP architecture on performance for the hippocampus segmentation experiment. The number of 3 × 3 × 3 convolutional layers and their associated number of learnable filters are specified in parentheses (e.g., [8–32] specifies eight 3 × 3 × 3 convolutional layers each with 32 filters)

	Dice (%)	MHD (mm]
CNN‐SP [8–32]	91.5 [2.0]	0.23 [0.08]
CNN‐SP [8–64]	91.4 [2.3]	0.24 [0.12]
CNN‐SP [10–32]	91.6 [2.0]	0.23 [0.07]
CNN‐SP [12–32]	91.7 [2.1]	0.23 [0.08]
CNN‐SP [14–32]	92.0 [1.8]	0.22 [0.06]
CNN‐SP [16–32]	91.9 [1.9]	0.21 [0.07]

*Note*: Mean Dice and MHD values over both left and right hippocampi are reported, with *SD*s in parentheses.

Abbreviations: CNN‐SP, CNN with spatial priors; MHD, modified Hausdorff distance.

#### 3.1.3 Effect of data augmentation

**Figure 4:**
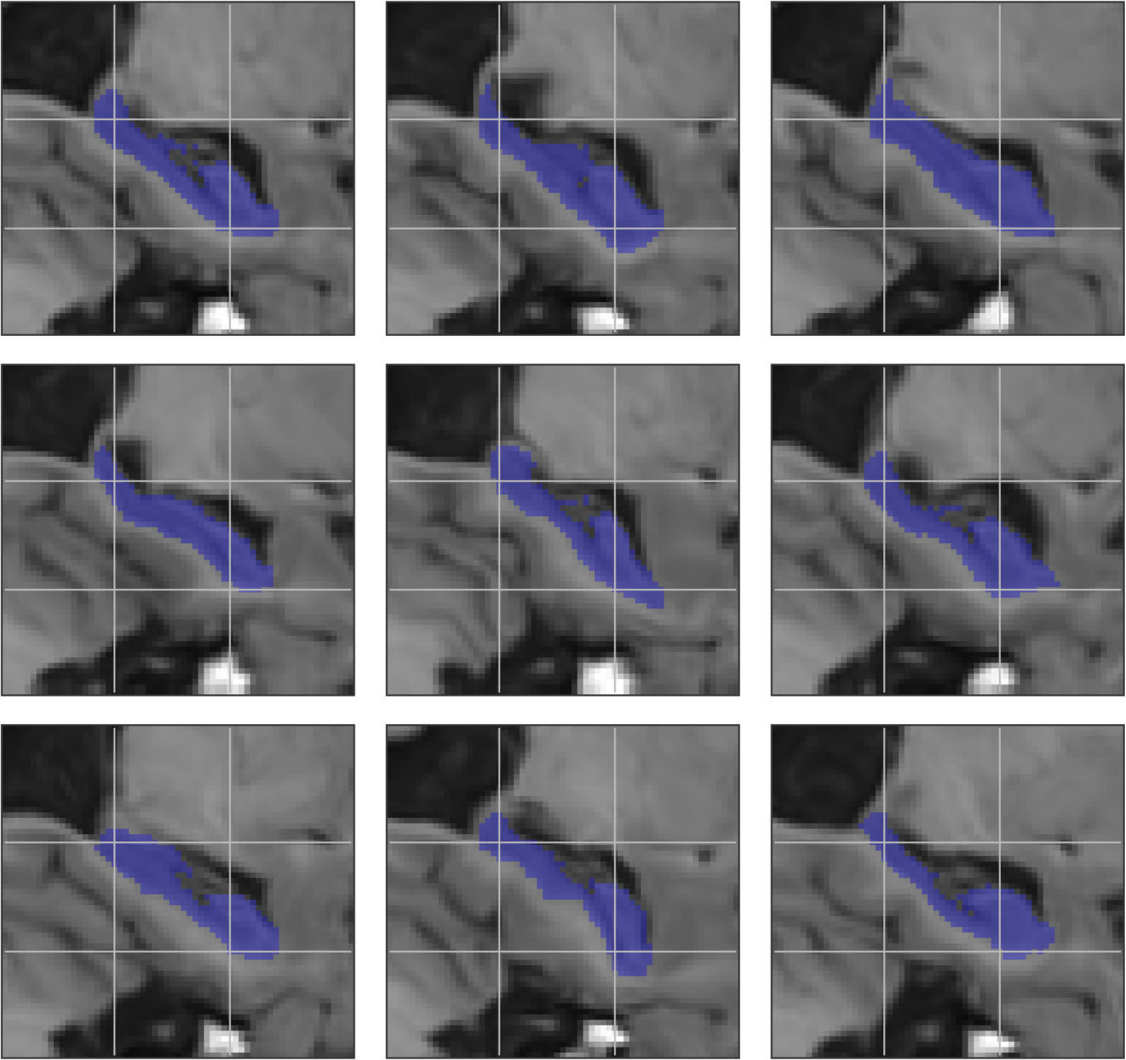
Example random deformations applied to an original sample (top left) using the parameters *σ*_*c*_ = 4 mm and *α*_*i*_ = 2 mm. A grid is overlaid on the samples to better highlight their differences [Color figure can be viewed at http://wileyonlinelibrary.com]

One concern when training deep CNNs with high modeling capacities is their increased tendency to overfit the training data, thus generalizing poorly when applied to new unseen testing data. As discussed in Section 2.3, data augmentation can be used to synthesize new training data to increase the generalizability of the trained networks. Using the CNN‐SP‐D architecture (i.e., twenty 3 × 3 × 3 convolutional layers, corresponding to input samples with spatial dimension 41^3^ voxels), we assessed the impact of random elastic deformation for data augmentation in two scenarios: the first, in which all available training subjects are used, and the second, in which only a randomly selected subset (25%, or 12 subjects) of the training subjects in each training fold is used, leaving the test folds unchanged. To determine the two parameters *σ*_*e*_ and *α*_*i*_ associated with our data augmentation scheme (see Section 2.3), we conducted a coarse‐grid search (applied in the second scenario) over *σ*_*e*_ = {4,8,16} mm and *α*_*i*_ = {1,2,4,8} mm, and found the best performance with *σ*_*e*_ = 4 mm and *α*_*i*_ = 2 mm. These parameters are used for data augmentation in the remaining experiments. Using these parameters, example randomly deformed training samples are shown in Figure 4.

Mean Dice and MHD values are reported in Table 4. As expected, the benefit of using data augmentation was largest when fewer training subjects were used: when using only 25% of the available training subjects (12 training subjects per fold), training the networks with data augmentation increased the mean Dice coefficient by 1.1% (*p* = 1 × 10^−11^) compared to training the networks without augmentation. The relative increase in mean Dice coefficient was reduced to only 0.2% (*p* = 0.06) when using all available training subjects. With regards to mean MHD, data augmentation resulted in improved performance only in the low‐data regime, reducing mean MHD from 0.28 to 0.24 mm (*p* = 1 × 10^−8^).

**Table 4:** Effect of DA with random elastic deformations on CNN‐SP‐D when using either 25% (12 training subjects per cross‐validation fold) or 100% (48 training subjects per cross‐validation subjects) of the available training data for the hippocampus segmentation experiment

		Dice (%)	MHD (mm)
25%	CNN‐SP‐D	90.0 (2.9)	0.28 (0.10)
	CNN‐SP‐D + DA	91.1 (2.1)	0.24 (0.08)
100%	CNN‐SP‐D	91.9 (1.9)	0.21 (0.07)
	CNN‐SP‐D + DA	92.1 (1.9)	0.21 (0.07)

*Note*: Mean Dice and MHD values over both left and right hippocampi are reported, with *SD*s in parentheses.

Abbreviations: DA, data augmentation; CNN‐SP, CNN with spatial priors; MHD, modified Hausdorff distance.

#### 3.1.4 Comparison to other methods

**Figure 5:**
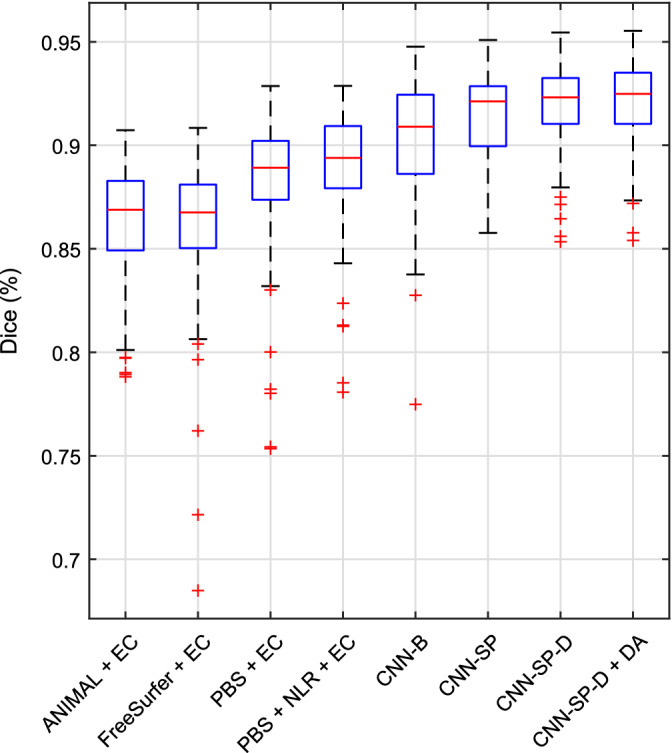
Boxplots of distributions of Dice coefficients (over both left and right hippocampi) from various methods for the segmentation of the left and right hippocampi in the Alzheimer's Disease Neuroimaging Initiative (ADNI) dataset [Color figure can be viewed at http://wileyonlinelibrary.com]

We further compared several variants of our CNN‐based method with several other popular and/or state‐of‐the‐art segmentation methods on the same dataset using the segmentations previously produced in the work of Zandifar et al. (2017), which includes results for four different methods, both before and after applying EC (Wang et al., 2011), a machine learning based wrapper which attempts to correct systematic errors made by the initial host segmentation method. The methods included are FreeSurfer (Fischl, 2012), ANIMAL (Collins & Pruessner, 2010) (a multiatlas technique combining nonlinear registration with majority‐vote label fusion), traditional PB segmentation (Coupé et al., 2011) (a multiatlas technique combining linear registration with PB label fusion), and an augmented approach combining PB segmentation with nonlinear registration. Mean Dice coefficients and MHD values are summarized in Table 5, and boxplots of distributions Dice coefficients are summarized in Figure 5.

**Table 5:** Comparison of four of our CNN‐based segmentation methods with previously reported results (Zandifar et al., 2017) for the segmentation of the left and right hippocampi in the ADNI dataset. Each table cell reports the mean Dice coefficient (*SD*) as a percentage on top and the mean MHD (*SD*), in millimeters, on bottom

	Left	Right	Both
CNN‐B	90.7 (2.3)	89.8 (3.2)	90.2 (2.8)
	0.25 (0.07)	0.29 (0.12)	0.27 (0.10)
CNN‐SP	91.5 (1.9)	91.6 (2.1)	91.5 (2.0)
	0.23 (0.07)	0.23 (0.09)	0.23 (0.08)
CNN‐SP‐D	92.0 (1.6)	91.8 (2.2)	91.9 (1.9)
	**0.21 (0.05)**	0.23 (0.08)	0.22 (0.07)
CNN‐SP‐D + DA	**92.0 (2.0)**	**92.2 (2.1)**	**92.1 (2.0)**
	0.22 (0.07)	**0.21 (0.07)**	**0.22 (0.08)**
ANIMAL	86.3 (2.6)	85.9 (3.0)	86.1 (2.8)
	0.40 (0.07)	0.42 (0.09)	0.41 (0.08)
ANIMAL + EC	86.5 (2.4)	86.2 (3.0)	86.4 (2.7)
	0.43 (0.08)	0.44 (0.09)	0.44 (0.08)
FreeSurfer	75.8 (4.7)	75.6 (4.8)	75.7 (4.7)
	0.94 (0.27)	0.98 (0.24)	0.96 (0.26)
FreeSurfer + EC	85.9 (3.3)	86.3 (3.1)	86.1 (3.2)
	0.44 (0.13)	0.42 (0.09)	0.43 (0.11)
PBS	87.5 (2.5)	87.3 (3.6)	87.4 (3.1)
	0.40 (0.09)	0.40 (0.13)	0.40 (0.11)
PBS + EC	88.2 (2.5)	88.2 (3.6)	88.2 (3.1)
	0.39 (0.08)	0.40 (0.14)	0.39 (0.11)
PBS + NLR	88.3 (2.2)	88.0 (3.2)	88.1 (2.7)
	0.39 (0.07)	0.39 (0.12)	0.39 (0.10)
PBS + NLR + EC	89.1 (2.6)	88.9 (3.1)	89.0 (2.6)
	0.37 (0.06)	0.37 (0.12)	0.37 (0.10)

*Note*: The top performing method is emboldened in each column.

Abbreviations: CNN, convolutional neural network; CNN‐B, baseline CNN; CNN‐SP, CNN with spatial priors; EC, error correction, NLR, nonlinear registration, PBS, patch‐based segmentation.

**Figure 6:**
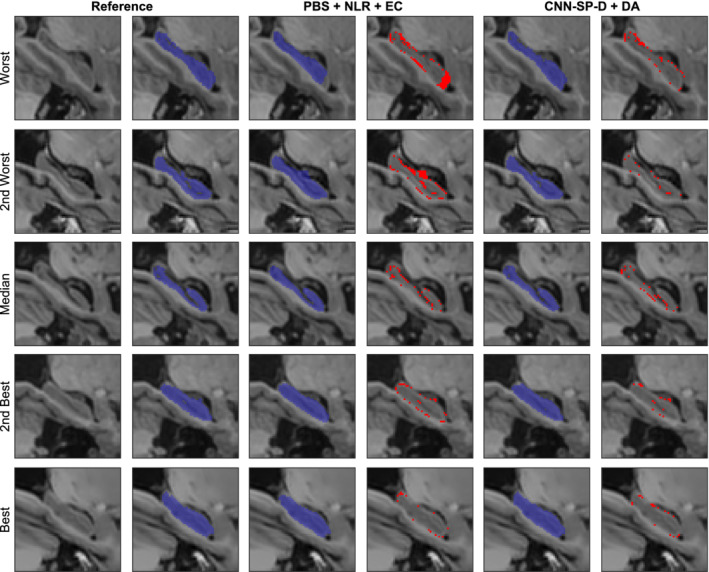
Example right hippocampus segmentations and respective errors using NLR + PB + EC and our best performing method CNN‐SP‐D + DA. The subjects with the worst, second worst, median, second best, and best overlap after applying NLR + PB + EC are shown for comparison. Errors are overlaid in red in columns four and six. EC, error correction; NLR, nonlinear registration; PBS, patch based; CNN‐SP, CNN with spatial priors [Color figure can be viewed at http://wileyonlinelibrary.com]

Compared to the best method from Zandifar et al. (2017), which combines PB segmentation with nonlinear registration and EC (PBS + NLR + EC), our best performing method (CNN‐SP‐D + DA) yielded an improvement of 2.1% in terms of mean Dice and a decrease in mean MHD of 0.17 mm (over both left and right hippocampi), both of which were highly statistically significant (*p* ≤ 10^−9^). CNN‐SP‐D + DA was also considerably more robust than the methods examined in the work of Zandifar et al., producing fewer outliers with low overlap (Figure 5). Example hippocampal segmentations comparing PBS + NLR + EC to CNN‐SP‐D + DA are displayed in Figure 6. While the more minor errors (occurring on the hippocampal boundary) made by both methods were similar, larger errors made by PBS + NLR + EC resulted in noncontiguous segmentations (e.g., first and second rows of Figure 6) that were avoided by CNN‐SP‐D + DA.

### 3.2 Applications to subcortical segmentation in IBSR dataset and multistructure segmentation in OASIS scan–rescan dataset

To further validate our method, we further applied it to the task of subcortical segmentation in the IBSR dataset, and multistructure segmentation in the OASIS scan–rescan dataset, in each case retraining, the networks on the respective datasets. Dataset details are provided in the respective sections below. We note that for the subsequent experiments, the network architecture is almost identical to that of the full‐proposed method used in the hippocampus segmentation experiments—only the number of output channels *C* (the number of classes) was changed for each respective segmentation task.

#### 3.2.1 Subcortical segmentation in the IBSR dataset

The IBSR dataset consists of 18 manually labeled T1w scans acquired at the Massachusetts General Hospital (http://www.cma.mgh.harvard.edu/ibsr/data.html). Of the 32 manually labeled structures, as done in Dolz, Desrosiers, and Ben Ayed (2017), we considered the left and right thalamus, caudate, putamen, and pallidum, for a total of nine classes (one class being background). Though the IBSR images are already roughly aligned, they differ in voxel sizes (ranging from 0.84 × 0.84 × 1.5 to 1 × 1 × 1.5 mm^3^) and would likely benefit from a finer‐grained registration and resampling. However, to demonstrate the robustness of our approach to small misalignments, we opted against this refinement step and used the images without additional pre‐processing. To obtain a segmentation for each subject, we carried out a sixfold cross‐validation (i.e., 15 training subjects and three test subjects per fold). We compared several variants of our method to the methods of Dolz, Desrosiers, and Ben Ayed (2017) and to a 2.5D CNN method (Kushibar et al. (2018)) that uses nonlinear registration to incorporate spatial probability maps. For the method of Dolz et al., we used their publicly available automated segmentations (https://github.com/josedolz/3D-F-CNN-BrainStruct/tree/master/Results/IBSR) to calculate performance measures. For the 2.5D CNN method, we include results exactly as reported in Kushibar et al., since the automated segmentations are not available for download. Lastly, we also include results from our own application of FIRST (Patenaude, Smith, Kennedy, & Jenkinson, 2011) from the FMRIB Software Library (Jenkinson, Beckmann, Behrens, Woolrich, & Smith, 2012).

**Figure 7:**
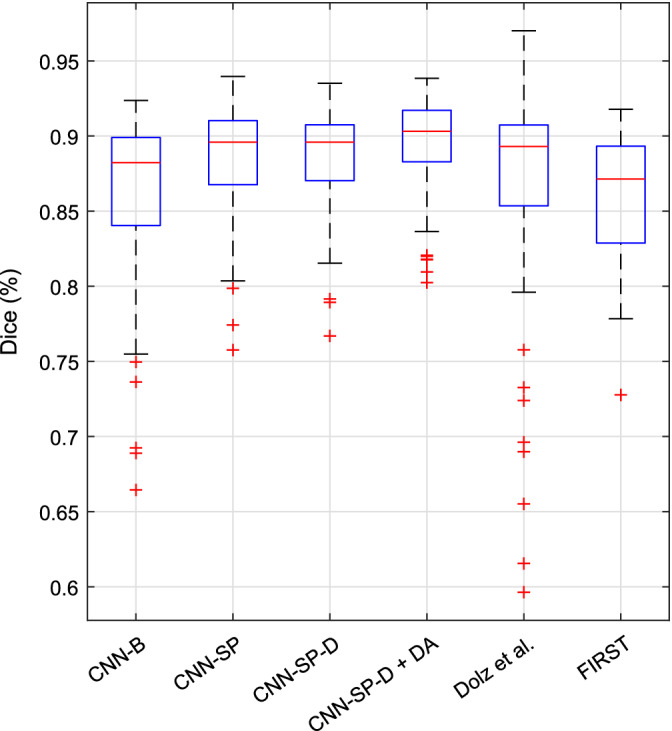
Boxplots of distributions of Dice coefficients (over all structures) from various methods for the segmentation subcortical structures in the IBSR dataset [Color figure can be viewed at http://wileyonlinelibrary.com]

Mean Dice coefficients and MHD values are summarized in Table 6, and boxplots of distributions of Dice coefficients are shown in Figure 7. Note that the results from the 2.5D CNN method (Kushibar et al., 2018) are not included in the boxplots because only summary statistics were available. Of our CNN‐based methods, the performance of the baseline network CNN‐B was poorest overall (mean Dice = 86.5% over all structures). Incorporating spatial priors, CNN‐SP produced a large (*p* = 9 × 10^−15^) increase in performance (mean Dice = 88.5%). Further deepening the network (CNN‐SP‐D) produced no significant increase in performance with respect to mean Dice (*p* = 0.76), which could be likely attributed to an increased capacity for overfitting due to the higher modeling capacity of the deeper network combined with the highly limited training data (i.e., only 15 subjects per cross‐validation fold) in this experiment. Indeed, regularizing the deeper network using data augmentation (CNN‐SP‐D + DA) produced a large (*p* = 2 × 10^−14^) increase in overlap (mean Dice = 89.5%) compared to CNN‐SP‐D. A similar pattern was observed with respect to mean MHD.

**Table 6:** Comparison of various segmentation methods for the segmentation of eight sub‐cortical structures in the IBSR dataset. Each table cell reports the mean Dice coefficient (*SD*) as a percentage on top and the mean MHD (*SD*), in millimeters, on bottom

	2.5D CNN	CNN‐B	CNN‐SP	CNN‐SP‐D	CNN‐SP‐D + DA	Dolz et al.	FIRST
L thalamus	91.0 (1.4)	88.3 (2.2)	90.8 (1.4)	90.2 (1.4)	**91.1 (1.2)**	90.1 (3.1)	89.9 (1.1)
	N/A	0.56 (0.13)	0.42 (0.09)	0.47 (0.10)	**0.42 (0.07)**	0.45 (0.17)	0.52 (0.06)
R thalamus	91.4 (1.6)	89.6 (1.9)	91.0 (1.4)	90.9 (1.5)	**91.5 (1.3)**	90.7 (2.8)	89.0 (1.3)
	N/A	0.58 (0.39)	0.43 (0.08)	0.43 (0.08)	**0.41 (0.07)**	0.44 (0.16)	0.55 (0.07)
L caudate	89.6 (1.8)	86.2 (5.1)	88.7 (3.8)	89.2 (2.4)	**89.9 (2.2)**	87.7 (6.4)	82.9 (2.6)
	N/A	0.37 (0.16)	0.28 (0.11)	0.27 (0.07)	**0.25 (0.06)**	0.39 (0.46)	0.41 (0.08)
R caudate	89.6 (2.0)	87.4 (5.4)	88.9 (3.0)	88.4 (2.6)	**90.0 (2.7)**	87.7 (7.3)	85.0 (4.7)
	N/A	0.35 (0.25)	0.29 (0.10)	0.31 (0.09)	**0.26 (0.10)**	0.33 (0.29)	0.34 (0.12)
L putamen	90.0 (1.4)	88.5 (2.7)	90.3 (1.5)	90.4 (1.4)	**91.0 (1.2)**	89.0 (4.5)	88.7 (1.4)
	N/A	0.37 (0.12)	0.32 (0.08)	0.32 (0.09)	**0.30 (0.07)**	0.38 (0.25)	0.41 (0.08)
R putamen	90.4 (1.2)	88.5 (3.0)	90.3 (1.6)	90.5 (1.5)	**91.6 (1.3)**	89.3 (5.4)	88.6 (1.1)
	N/A	0.37 (0.12)	0.32 (0.09)	0.32 (0.09)	**0.27 (0.06)**	0.39 (0.35)	0.42 (0.08)
L pallidum	82.6 (5.0)	82.0 (3.9)	83.9 (2.6)	84.5 (2.8)	**85.5 (2.2)**	82.6 (5.7)	82.3 (2.4)
	N/A	0.47 (0.14)	0.42 (0.12)	0.40 (0.12)	**0.38 (0.09)**	0.44 (0.18)	0.42 (0.09)
R pallidum	82.9 (4.6)	81.4 (6.1)	84.0 (2.8)	84.4 (3.0)	**85.5 (2.7)**	83.1 (6.3)	82.8 (2.6)
	N/A	0.49 (0.21)	0.41 (0.12)	0.40 (0.12)	**0.38 (0.12)**	0.43 (0.17)	0.41 (0.09)
All	N/A	86.5 (4.9)	88.5 (3.6)	88.6 (3.2)	**89.5 (3.1)**	87.5 (6.0)	86.2 (3.8)
	N/A	0.45 (0.22)	0.36 (0.12)	0.37 (0.11)	**0.33 (0.11)**	0.41 (0.27)	0.44 (0.10)

*Note*: The top performing method is emboldened in each row.

Abbreviations: CNN‐B, baseline CNN; CNN‐SP, CNN with spatial priors; MHD, modified Hausdorff distance.

Comparing our methods to those from other works, our best performing method without data augmentation (CNN‐SP‐D) performed similarly to the 2.5D CNN approach (Kushibar et al., 2018), achieving segmentations with slightly better overlap in the putamen and pallidum, and slightly worse overlap in the thalamus and caudate. However, we CNN‐SP‐D does not depend on expensive nonlinear registration, making it considerably faster (16.4 ± 2.0 s per subject) compared to the 2.5D CNN approach (approximately 5 min per subject). The method of Dolz et al. performed better than our baseline method CNN‐B with respect to both mean Dice (*p* = 3 × 10^−4^) and mean MHD (*p* = 2 × 10^−4^). Compared to the method of Dolz et al., both of our CNN‐based methods incorporating spatial priors (CNN‐SP and CNN‐SP‐D) resulted in significant increases with respect to mean Dice (*p* < 0.01) but not with respect to mean MHD (*p* > 0.21). Finally, combining CNN‐SP‐D with data augmentation resulted in the best performance out of the six CNN‐based methods with respect to both mean Dice and mean MHD (*p* < 2 × 10^−11^). However, we note that the data augmentation scheme used in this work is general and could be used to also boost the performance of the other CNN‐based methods under comparison.

**Figure 8:**
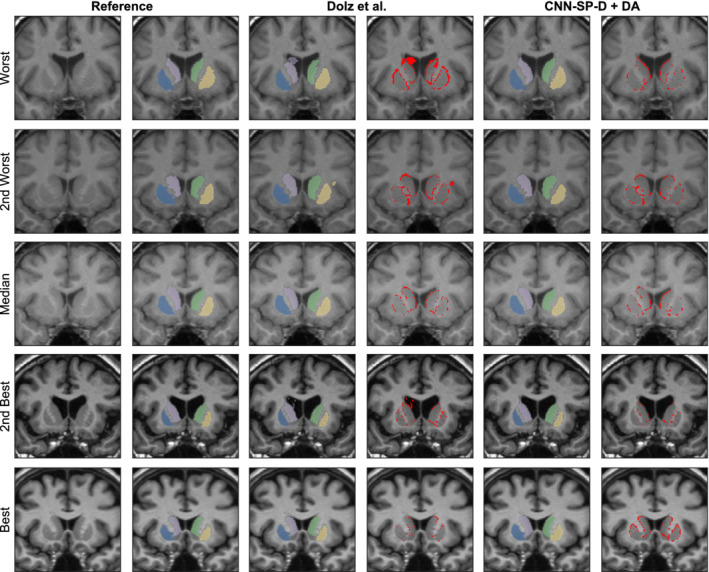
Example subcortical segmentations and respective errors using the method of Dolz, Desrosiers, and Ben Ayed (2017) and our best performing method CNN‐SP‐D + DA. The subjects with the worst, second worst, median, second best and best overlap after applying the method of Dolz et al. are shown for comparison. The putamen is overlaid in yellow and blue, and the caudate in light purple and green. Errors are overlaid in red in columns four and six [Color figure can be viewed at http://wileyonlinelibrary.com]

As shown in Figure 7, CNN‐SP‐D + DA produced fewer outliers with low overlap coefficients when compared to CNN‐B and the method of Dolz et al., two similar CNN‐based methods which do not exploit spatial priors. Example segmentations comparing the approach of Dolz et al. to CNN‐SP‐D + DA are shown in Figure 8. Our method produced more contiguous segmentations, for example, better avoiding irregular expansions of the segmentation into the surrounding lateral ventricle (first and fourth rows of Figure 8) and better avoiding small clusters of false positives (second row of Figure 8).

#### 3.2.2 Multistructure segmentation in the OASIS scan–rescan dataset

The OASIS (Marcus et al., 2007) scan–rescan dataset contains T1w scans of 20 healthy young adult subjects each scanned on two separate occasions within a period of 90 days. The images were acquired on a 1.5 T Siemens Vision scanner using an MP‐RAGE acquisition sequence, and have a size of 256 × 256 × 128 voxels with a voxel size of 1 × 1 × 1.25 mm^3^. Expert manually generated labels of both session images are available by subscription to Neuromorphometrics (http://www.neuromorphometrics.com). All CNN‐based methods used pre‐processing consisting of N3 nonuniformity correction and affine registration to the MNI‐ICBM152 template with 1 × 1 × 1 mm^3^ resolution. All registrations for this experiment were carried out using a multiscale registration algorithm with a normalized mutual information similarity measure, based on the MINC toolkit (https://github.com/bic-mni) and detailed in Dadar, Fonov, and Collins (2018).

To obtain a segmentation for each subject, we carried out a fivefold cross validation experiment (i.e., 16 training subjects and four testing subjects per fold) using the first session images. For each fold, we additionally applied the trained networks to the same test subjects belonging to the second imaging session. All label estimates were then resampled back to native space, using nearest‐neighbor interpolation, with the inverse of the corresponding transform estimated during the preprocessing stage.

Accuracy (i.e., agreement with manual labels) was assessed using a fivefold cross validation (i.e., 16 training subjects and four testing subjects per fold) on images from the first session only using the manual labels as the reference labels. To estimate the scan–rescan reliability of the different labeling methods, second‐session images (and their corresponding labels) were registered (by estimating a six‐parameter rigid transformation) to the corresponding first‐session images, and the consistency across label pairs was assessed using both the Dice coefficient and the MHD measures.

While the manual labelings for this dataset were carried out in accordance with a strict protocol, no special effort was made to make the boundaries between regions as smooth as possible. In preliminary studies, we observed that the manual reliability estimates appeared artificially low because of these noisy boundaries. We therefore smoothed the manual labels using median filtering with a small 3 × 3 × 3 kernel, which resulted in higher and more reasonable manual reliability estimates. For completeness, we also performed the same analysis below but using the original unfiltered manual segmentations as reference labels (see supplementary material, Tables S1 and S2).

**Figure 9:**
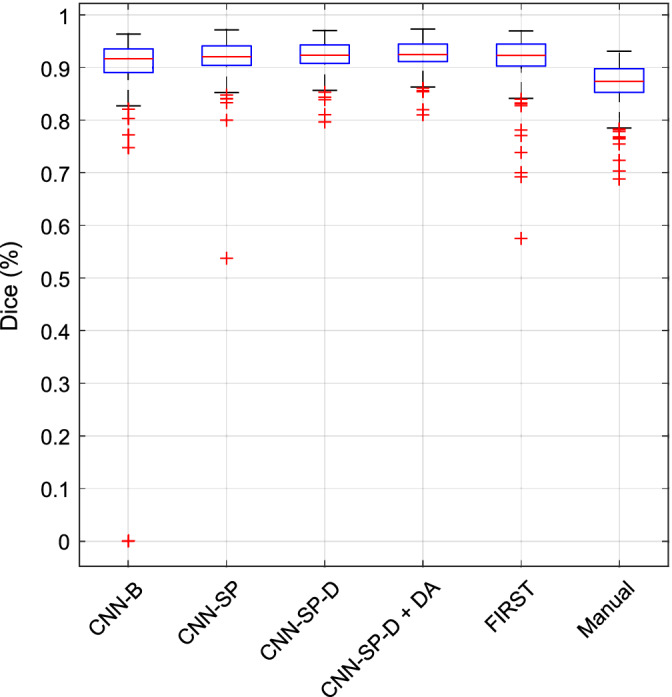
Reliability in OASIS scan–rescan dataset. Boxplots of distributions of Dice coefficients (over all structures) of various methods for are plotted [Color figure can be viewed at http://wileyonlinelibrary.com]

The scan–rescan reliabilities of manual labeling as well as the automated methods under comparison are reported in Table 7, and boxplots of distributions of Dice coefficients are shown in Figure 9. Each of the automated methods under comparison produced more reliable segmentations compared to manual segmentation (mean Dice = 86.9%, mean MHD = 0.39 mm, over all structures). FIRST was comparably reliable (mean Dice = 91.7%, mean MHD = 0.24 mm) to CNN‐SP, and more reliable compared to CNN‐B (mean Dice = 90.7%, mean MHD = 0.44 mm). Both CNN‐SP‐D and CNN‐SP‐D + DA produced the most reliable segmentations (mean Dice ≥92.2%, mean MHD ≤0.21 mm).

**Table 7:** Reliability in the OASIS scan–rescan dataset. Each table cell reports the mean Dice coefficient (*SD*) as a percentage on top and the mean MHD (*SD*), in millimeters, on bottom

	CNN‐B	CNN‐SP	CNN‐SP‐D	CNN‐SP‐D + DA	FIRST	Manual
L caudate	92.4 (1.5)	92.1 (1.8)	92.7 (1.5)	**92.7 (1.5)**	90.8 (5.0)	87.2 (2.3)
	0.18 (0.03)	0.19 (0.05)	0.18 (0.03)	**0.17 (0.03)**	0.24 (0.14)	0.33 (0.06)
R caudate	91.9 (1.2)	91.8 (1.2)	92.5 (0.9)	**92.5 (0.8)**	91.9 (0.9)	87.5 (2.4)
	0.22 (0.14)	0.19 (0.03)	**0.18 (0.02)**	0.18 (0.02)	0.20 (0.02)	0.33 (0.11)
L putamen	93.3 (1.5)	93.7 (1.4)	94.0 (1.2)	93.9 (1.2)	**94.5 (0.6)**	88.9 (2.2)
	0.20 (0.04)	0.19 (0.04)	0.19 (0.04)	**0.18 (0.04)**	0.20 (0.02)	0.35 (0.09)
R putamen	93.2 (1.2)	93.5 (1.4)	94.0 (0.8)	93.9 (0.9)	**94.6 (0.5)**	89.1 (2.0)
	0.21 (0.04)	0.20 (0.04)	**0.19 (0.03)**	0.19 (0.03)	0.20 (0.02)	0.35 (0.08)
L thalamus	94.5 (1.7)	95.3 (0.9)	95.4 (0.8)	95.5 (0.7)	**96.1 (0.7)**	91.5 (0.7)
	0.37 (0.59)	0.21 (0.04)	0.20 (0.03)	0.20 (0.03)	**0.20 (0.03)**	0.41 (0.04)
R thalamus	94.8 (0.8)	95.3 (0.8)	95.5 (0.6)	95.5 (0.6)	**95.9 (0.6)**	91.8 (0.9)
	0.33 (0.32)	0.21 (0.04)	**0.20 (0.03)**	0.21 (0.03)	0.21 (0.03)	0.40 (0.06)
L hippocampus	89.2 (2.0)	90.9 (1.5)	91.1 (1.2)	**91.5 (1.2)**	90.8 (1.3)	86.6 (1.1)
	0.24 (0.05)	0.22 (0.04)	0.21 (0.03)	**0.20 (0.02)**	0.24 (0.05)	0.34 (0.04)
R hippocampus	89.0 (3.3)	90.1 (1.8)	91.0 (1.1)	**91.2 (0.9)**	91.1 (0.8)	86.2 (1.1)
	0.37 (0.61)	0.24 (0.07)	0.21 (0.03)	**0.20 (0.02)**	0.22 (0.02)	0.36 (0.06)
L pallidum	91.3 (2.3)	92.1 (1.7)	92.2 (1.7)	**92.8 (1.4)**	92.3 (1.9)	85.5 (4.7)
	0.23 (0.05)	0.21 (0.05)	0.21 (0.05)	**0.19 (0.04)**	0.22 (0.05)	0.43 (0.14)
R pallidum	90.5 (1.7)	91.7 (2.0)	92.1 (1.0)	**92.4 (1.2)**	91.2 (2.9)	85.7 (3.6)
	0.24 (0.04)	0.22 (0.07)	0.20 (0.03)	**0.20 (0.04)**	0.26 (0.10)	0.41 (0.12)
L amygdala	82.0 (19.1)	87.7 (2.7)	88.1 (2.8)	**88.6 (2.7)**	84.8 (7.2)	80.8 (4.5)
	2.37 (8.90)	0.26 (0.05)	0.26 (0.06)	**0.24 (0.05)**	0.34 (0.15)	0.48 (0.16)
R amygdala	86.5 (3.1)	86.3 (7.9)	87.6 (2.8)	**88.7 (2.4)**	85.8 (5.2)	81.4 (2.4)
	0.28 (0.07)	0.31 (0.22)	0.27 (0.06)	**0.25 (0.05)**	0.32 (0.14)	0.44 (0.08)
All	90.7 (6.8)	91.7 (3.8)	92.2 (2.9)	**92.4 (2.6)**	91.7 (4.7)	86.9 (4.2)
	0.44 (2.65)	0.22 (0.09)	0.21 (0.05)	**0.20 (0.04)**	0.24 (0.09)	0.39 (0.11)

*Note*: The top performing method is emboldened in each row.

Abbreviations: CNN‐B, baseline CNN; CNN‐SP, CNN with spatial priors; MHD, modified Hausdorff distance.

**Figure 10:**
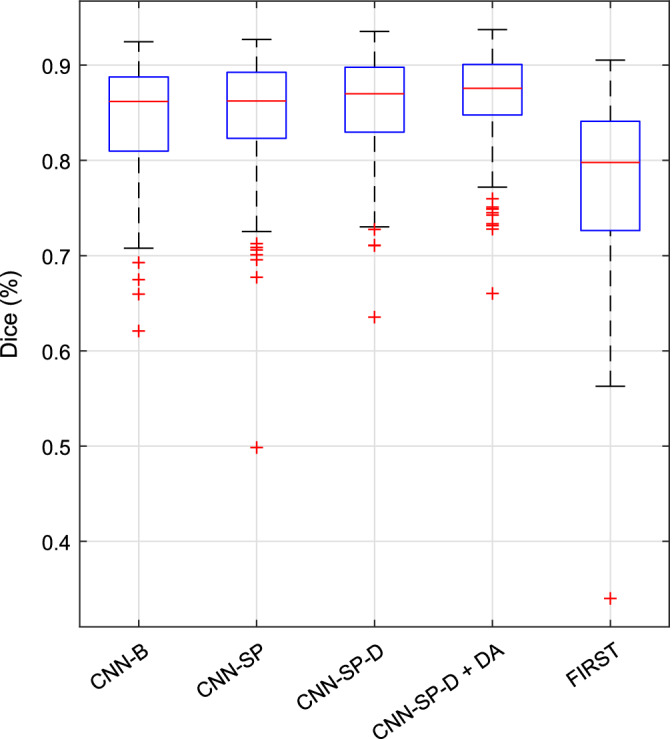
Accuracy in OASIS scan–rescan dataset. Boxplots of distributions of Dice coefficients (over all structures) of various methods are plotted [Color figure can be viewed at http://wileyonlinelibrary.com]

The accuracy of the automated methods under comparison is reported in Table 8, and boxplots of distributions of Dice coefficients are shown in Figure 10. Among the CNN‐based methods, CNN‐B (mean Dice = 84.6%, mean MHD = 0.50 mm over all structures) again performed poorest overall, followed by CNN‐SP (mean Dice = 85.0%, mean MHD = 0.48 mm). It is worth noting that in this dataset, augmenting the baseline network with spatial priors provided a relatively minor performance gain compared to the previous segmentation tasks. Using a deeper network (CNN‐SP‐D), however, proved particularly beneficial for this task (mean Dice = 86.0%, mean MHD = 0.42 mm). Finally, CNN‐SP‐D was further improved using data augmentation (CNN‐SP‐D + DA), resulting in the best performance (mean Dice = 86.6%, mean MHD = 0.41 mm). A Wilcoxon signed‐rank test between the accuracy of CNN‐SP‐D + DA and the reliability of manual labelings (mean Dice = 86.9%, mean MHD = 0.39 mm) showed no significant difference with respect to mean Dice (*p* = 0.72); however, the mean MHD of manual labelings was slightly but significantly lower (*p* = 0.02) compared to CNN‐SP‐D + DA.

**Table 8:** Accuracy in the OASIS scan–rescan dataset. Each table cell reports the mean Dice coefficient (*SD*) as a percentage on top and the mean MHD (*SD*), in millimeters, on bottom

	CNN‐B	CNN‐SP	CNN‐SP‐D	CNN‐SP‐D + DA	FIRST
L caudate	85.7 (3.8)	85.4 (3.7)	86.5 (3.0)	**87.0 (2.8)**	71.3 (6.3)
	0.45 (0.25)	0.44 (0.16)	**0.36 (0.09)**	0.37 (0.11)	0.94 (0.22)
R caudate	86.5 (3.2)	85.8 (4.0)	87.1 (3.0)	**87.1 (2.6)**	68.4 (7.2)
	0.42 (0.22)	0.42 (0.33)	**0.34 (0.09)**	0.34 (0.08)	1.00 (0.25)
L putamen	88.1 (2.3)	88.5 (3.0)	89.2 (3.0)	**89.3 (2.6)**	84.7 (2.1)
	0.41 (0.10)	0.37 (0.11)	0.35 (0.11)	**0.34 (0.10)**	0.60 (0.09)
R putamen	88.4 (1.9)	88.6 (1.9)	89.3 (2.1)	**89.4 (2.0)**	84.2 (2.5)
	0.38 (0.07)	0.38 (0.07)	0.36 (0.08)	**0.35 (0.07)**	0.64 (0.11)
L thalamus	89.5 (1.6)	89.5 (2.2)	90.4 (2.1)	**90.5 (1.7)**	86.1 (2.3)
	0.55 (0.09)	0.55 (0.17)	0.48 (0.13)	**0.47 (0.10)**	0.79 (0.13)
R thalamus	90.5 (1.6)	90.8 (1.9)	**91.8 (1.2)**	91.6 (1.2)	88.0 (1.3)
	0.65 (0.57)	0.49 (0.24)	**0.41 (0.07)**	0.43 (0.07)	0.68 (0.08)
L hippocampus	81.4 (5.8)	84.3 (3.2)	85.2 (2.0)	**86.9** (1.5)	80.0 (1.8)
	0.52 (0.22)	0.51 (0.35)	0.40 (0.09)	**0.34 (0.06)**	0.60 (0.07)
R hippocampus	83.1 (3.5)	83.9 (3.8)	85.7 (2.0)	**86.2 (1.6)**	79.7 (2.1)
	0.44 (0.13)	0.55 (0.59)	**0.39 (0.10)**	0.39 (0.08)	0.60 (0.07)
L pallidum	83.7 (3.4)	83.7 (4.4)	83.7 (4.5)	**84.6 (4.4)**	77.3 (6.2)
	0.50 (0.12)	0.51 (0.16)	0.51 (0.16)	**0.47 (0.16)**	0.78 (0.26)
R pallidum	82.8 (4.3)	84.7 (4.3)	84.9 (4.1)	**86.0 (3.8)**	76.3 (6.5)
	0.50 (0.15)	0.46 (0.16)	0.45 (0.14)	**0.42 (0.13)**	0.79 (0.24)
L amygdala	76.6 (5.1)	77.6 (5.0)	77.2 (5.4)	**79.1 (5.4)**	68.8 (9.2)
	0.62 (0.32)	0.54 (0.13)	0.58 (0.17)	**0.53 (0.17)**	0.98 (0.37)
R amygdala	78.8 (4.1)	77.6 (7.4)	80.6 (2.9)	**81.7 (3.6)**	70.6 (5.0)
	0.50 (0.15)	0.57 (0.23)	0.47 (0.09)	**0.45 (0.11)**	0.85 (0.16)
All	84.6 (5.5)	85.0 (5.7)	86.0 (5.1)	**86.6 (4.6)**	78.0 (8.4)
	0.50 (0.25)	0.48 (0.27)	0.42 (0.13)	**0.41 (0.13)**	0.77 (0.24)

*Note*: The top performing method is emboldened in each row.

Abbreviations: CNN‐B, baseline CNN; CNN‐SP, CNN with spatial priors; MHD, modified Hausdorff distance.

**Figure 11:**
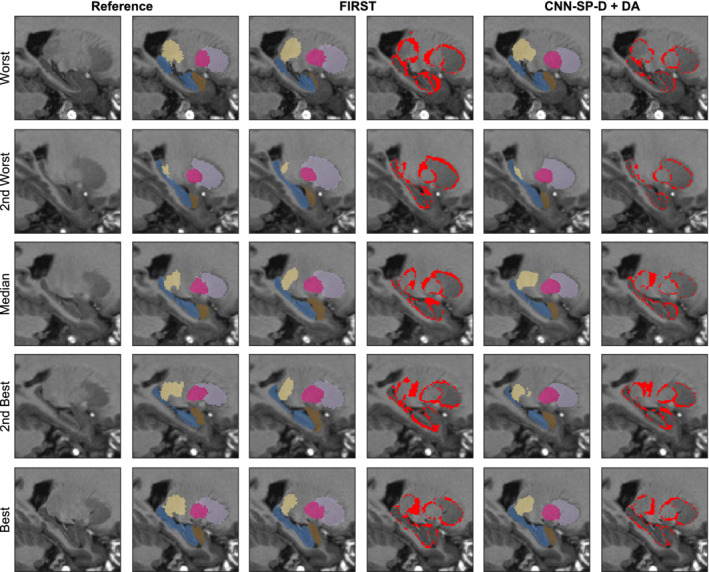
Example multistructure segmentations and respective errors using FIRST and our best performing method CNN‐SP‐D + DA. The subjects with the worst, second worst, median, second best, and best overlap after applying FIRST are shown for comparison. The hippocampus is overlaid in blue, amygdala in brown, thalamus in yellow, putamen in pink, and caudate in light purple. Errors are overlaid in red in columns four and six [Color figure can be viewed at http://wileyonlinelibrary.com]

Despite FIRST being a highly reliable method, it was overall the least accurate method under comparison (mean Dice = 78.0%, mean MHD = 0.77 mm, over all structures). This is likely because FIRST does not learn from user‐specified training data, but instead incorporates priors derived from its own set of training data, which may differ with respect to the anatomical protocol used for manual labeling. Example segmentations comparing FIRST to our best performing CNN‐based method are shown in Figure 11. Both FIRST and CNN‐SP‐D + DA produced very smooth labelings; however, FIRST had more difficulty in delineating the caudate (which tended to be undersegmented) and the amygdala. We also note that the error counts (e.g., the fourth and sixth columns of Figure 11) were larger in this experiment compared to the previous two, particularly along structure boundaries. We believe that this can be largely attributed to noisy manual segmentations.

## 4 DISCUSSION

While many of the other segmentation methods compared in this work also performed reasonably well in general, we demonstrated in a series of three validation experiments that our proposed method consistently produced segmentations with higher accuracy and robustness (i.e., fewer outliers). Using a scan–rescan dataset, we further demonstrated that the proposed method is highly reliable and produces segmentations with an accuracy comparable to that of the scan–rescan reliability of expert manual segmentations. Finally, our proposed method maintains the highly competitive runtime performance common among many recent CNN‐based methods for segmentation (Dolz, Desrosiers, & Ben Ayed, 2017; Guha Roy et al., 2018; Kushibar et al., 2018). While the short runtimes associated with these recent CNN‐based methods (requiring seconds or minutes as opposed to hours for some multi‐atlas segmentation methods (e.g., Asman & Landman, 2013; Wang & Yushkevich, 2013) may not be of crucial importance for some tasks, runtimes on the orders of seconds allows for the processing of large datasets on single GPU workstations instead of computing clusters, and may be of particular clinical importance for intraoperative applications.

Because of the high degree of regularity in the location of many neuroanatomical structures when normalized to a common space, spatial context is a powerful tool to exploit in MRI segmentation. In Section 3.1.1, it was demonstrated that using spatial priors to assist CNN‐based segmentation not only improves performance, but also significantly reduces the computation time required for applying a trained CNN. While a major advantage of deep‐learning methods over traditional multi‐atlas segmentation is their reduced reliance on extensive image preprocessing, our method only requires linear registration to a common space, which is fast and in many cases necessary for subsequent processing steps. While the specific choice of linear registration algorithm may not be of crucial importance, due to our methods reliance on spatial priors, we expect that failed registrations would result in correspondingly poor segmentations. However, we note that linear registration can be highly robust: Dadar et al. (2018) report a failure rate of less than 0.5% using our preferred registration tool, publicly available as part of the MINC toolkit.

Further performance gains could possibly be obtained by using nonlinear registration to a common template: the use of nonlinear registration would, in ideal circumstances, produce more restrictive working volumes (further reducing processing time when applying a trained CNN), and increase the predictive power of spatial coordinates. However, the use of nonlinear registration introduces several practical complications: traditional nonlinear registration is extremely computationally expensive relative to the time required to apply a trained CNN, and study‐specific templates are often required for robust nonlinear registration. Combining our approach with deep‐learning approaches for nonlinear image registration, which have potential for much better computational efficiency, may be a promising avenue for future work. We emphasize however that any performance gains due to the use of spatial priors are to be expected only in proportion to the spatial regularity of the structure of interest. For example, it would not be helpful to use either a working volume or spatial coordinates for the segmentation of brain tumors, which are highly heterogeneous in shape, size, appearance, and location.

Deep networks like the ones used in this work have a high modeling capacity and are therefore can be more prone to overfitting, particularly when few training samples are available. Indeed this is commonly the case for tasks such as neuroanatomical segmentation, where generating large quantities of high quality training data is a very tedious and time‐consuming task. While subsampling a volume into smaller subvolumes (patches) is effectively a form of data augmentation, many of the patches extracted from or nearby a particular structure of a given subject will overlap to a large extent (particularly for small structures) and will therefore be somewhat redundant. Overfitting is therefore still possible (as observed in Section 3.1.2), making more aggressive data augmentation schemes necessary for training networks with good generalizability. While many other techniques have been proposed to deal with limited training data (e.g., fine‐tuning networks pretrained on automatic segmentations as done in Guha Roy et al. (2018)), we demonstrated excellent performance using a data augmentation scheme based on random elastic deformations. More advanced deformation‐based techniques could be also investigated, for example, learning a more limited space of plausible deformations using statistical modeling techniques (Hauberg, Freifeld, Larsen, Fisher, & Hansen, 2016; Onofrey, Papademetris, & Staib, 2015), generating random topology‐preserving diffeomorphisms, or a combination of both. Assessing the impact of more realistic transformations for data augmentation is a promising research direction which we leave to future work.

Further contributing to the good performance of the proposed method in cases of very limited training data is the relatively low number of parameters associated with our networks (~5 × 10^5^ parameters in our deep network, limiting the capacity to overfit (for comparison, we note that the original U‐Net architecture (Ronneberger et al., 2015) has ~2 × 10^7^ parameters). This is in large part due to our choice of using only 32 learnable convolutional filters per layer, since widening the networks showed no appreciable improvement in performance (see Section 3.1.2). On the other hand, increasing the depth of the network (and correspondingly increasing the size of the input patch) resulted in considerable performance gains, which can be attributed to the increased spatial context available to the network in addition to a much higher modeling capacity. Indeed, it has been demonstrated that making networks deeper, as opposed to wider, is a more parameter‐efficient way of increasing the modeling capacity of a network (Eldan & Shamir, 2016). While it is likely that the performance of our network could be further improved by fine‐tuning the network architecture for specific segmentation tasks, we opted against such an approach to highlight the versatility of this particular network architecture.

A related problem concerns that of generalization across datasets. Since the learned convolutional layers (particularly deeper into the network (Ghafoorian et al., 2017)) are highly tailored to the peculiarities of the training data, it is commonly the case that networks trained on a certain dataset perform poorly when applied to an unseen dataset. Nonetheless, robustness to differences between training and testing images (e.g., due to differences in age, health, scanner type, field strength, and/or acquisition sequence) is a highly desirable quality of any method for MRI segmentation. While the ADNI and IBSR datasets considered in this work are highly heterogeneous, still more challenging scenarios are commonly encountered in practice. For example, given the often prohibitively high cost of generating high quality manual labelings, it may be desirable to apply a trained classifier to images do not have an adequate representation whatsoever in the training set. Future work will address this problem by leveraging so‐called “domain adaptation” methods (e.g., Ganin et al., 2016; Hoffman, Wang, Yu, & Darrell, 2016) to learn networks which are robust to differences between the training and target image domains, further increasing the general applicability of our approach.

## Supporting information


**Appendix S1** Supporting Information.

## Data Availability

In the spirit of open science, all data used to generate the tables and figures shown in this work, as well as the code used to train and test the networks, will be made publicly available at https://github.com/philnovv/CNN_NeuroSeg.
