# Supplementary material for: Accurate and robust segmentation of neuroanatomy in T1‐weighted MRI by combining spatial priors with deep convolutional neural networks
Source: Hum Brain Mapp. 2019 Oct 21;41(2):309–27. doi: 10.1002/hbm.24803 (PMC7267949; doi:10.1002/hbm.24803)
Supplement: Supplementary file 1 — Appendix S1 Supporting Information. [file HBM-41-309-s001.pdf]

Accurate and robust segmentation of neuroanatomy in T1-weighted MRI by combining spatial priors with deep convolutional neural networks

Philip Novosad[a,b,*], Vladimir Fonov[a,b], D. Louis Collins[a,b], and Alzheimer's Disease Neuroimaging Initiative[1]

[*]Corresponding author at: McConnell Brain Imaging Centre, Montreal Neurological Institute, McGill University, Montreal, Canada. E-mail address: philip.novosad@mail.mcgill.ca

[a]McConnell Brain Imaging Centre, Montreal Neurological Institute, McGill University, Montreal, Canada

[b]Department of Biomedical Engineering, McGill University, Montreal, Canada

[1]Data used in preparation of this article were obtained from the Alzheimer's Disease Neuroimaging Initiative (ADNI) database (adni.loni.usc.edu). As such, the investigators within the ADNI contributed to the design and implementation of ADNI and/or provided data but did not participate in analysis or writing of this report. A complete listing of ADNI investigators can be found at http://adni.loni.usc.edu/wp-content/uploads/how_to_apply/ADNI_Acknowledgement_List.pdf.

1 Supplementary material

Tables 9 and 10 summarize the reliability and accuracy of the compared segmentation methods in the OASIS scan-rescan dataset using the original manual reference segmentations provided by Neuromorphometrics (i.e. without median filtering). All networks were re-trained on the original labels, and all performance metrics in Table 10 were re-calculated using the original unfiltered labels as the references. Training on the original labels had little effect on the reproducibility of the CNN-based methods. However, using the original manual labels resulted in a drop in the reliability estimates of manual labelling, from a mean Dice of 86.9% over all structures (using median filtering) to a mean Dice of 83.9% ($p = 4 \times 10^{-41}$). The accuracy of the various automated methods also dropped when using the original manual segmentations as the reference. For example, the mean Dice coefficient of the top performing method CNN-SP-D + DA dropped from 86.6% over all structures to 85.1% ($p = 1 \times 10^{-27}$).

	CNN-B	CNN-SP	CNN-SP-D	CNN-SP-D + DA	FIRST	Manual
L Caudate	92.4 (1.4)	92.5 (1.6)	92.8 (1.4)	**92.9 (1.4)**	90.8 (5.0)	85.1 (2.2)
	0.19 (0.03)	0.19 (0.04)	0.17 (0.03)	**0.17 (0.03)**	0.24 (0.14)	0.35 (0.06)
R Caudate	92.0 (1.0)	92.1 (0.9)	92.2 (0.9)	**92.4 (0.9)**	91.9 (0.9)	85.2 (2.3)
	0.19 (0.02)	0.19 (0.03)	0.18 (0.02)	**0.18 (0.02)**	0.20 (0.02)	0.34 (0.06)
L Putamen	92.9 (1.5)	93.7 (1.3)	93.9 (1.2)	94.0 (1.2)	**94.5 (0.6)**	86.1 (1.9)
	0.21 (0.04)	0.19 (0.05)	0.19 (0.04)	**0.18 (0.04)**	0.20 (0.02)	0.39 (0.07)
R Putamen	93.2 (1.2)	93.7 (1.1)	93.9 (0.9)	94.0 (0.8)	**94.6 (0.5)**	86.4 (1.7)
	0.21 (0.04)	0.20 (0.04)	0.19 (0.03)	**0.19 (0.02)**	0.20 (0.02)	0.39 (0.06)
L Thalamus	94.7 (1.2)	95.0 (0.8)	95.4 (0.8)	95.5 (0.7)	**96.1 (0.7)**	89.6 (0.6)
	0.24 (0.06)	0.22 (0.04)	0.21 (0.03)	0.20 (0.03)	**0.20 (0.03)**	0.44 (0.04)
R Thalamus	95.0 (0.8)	95.3 (0.8)	95.5 (0.8)	95.5 (0.7)	**95.9 (0.6)**	89.7 (0.9)
	0.23 (0.04)	0.22 (0.04)	**0.21 (0.04)**	0.21 (0.03)	0.21 (0.03)	0.44 (0.05)
L Hippocampus	89.5 (1.6)	90.5 (1.6)	91.2 (1.1)	**91.6 (1.0)**	90.8 (1.3)	83.3 (0.9)
	0.24 (0.04)	0.24 (0.07)	0.21 (0.03)	**0.20 (0.03)**	0.24 (0.05)	0.38 (0.03)
R Hippocampus	88.9 (1.8)	90.2 (1.5)	90.8 (1.4)	**91.2 (1.2)**	91.1 (0.8)	82.5 (1.0)
	0.24 (0.04)	0.23 (0.04)	0.22 (0.03)	**0.21 (0.02)**	0.22 (0.02)	0.41 (0.06)
L Pallidum	90.5 (3.1)	91.9 (1.8)	92.0 (1.9)	**92.5 (1.6)**	92.3 (1.9)	81.9 (3.9)
	0.24 (0.07)	0.21 (0.04)	0.21 (0.05)	**0.20 (0.05)**	0.22 (0.05)	0.47 (0.11)
R Pallidum	90.1 (2.6)	91.9 (1.4)	**92.2 (1.1)**	92.1 (1.2)	91.2 (2.9)	82.1 (3.3)
	0.25 (0.06)	0.21 (0.04)	**0.20 (0.03)**	0.21 (0.03)	0.26 (0.10)	0.46 (0.09)
L Amygdala	84.1 (4.1)	85.9 (4.1)	87.7 (2.3)	**88.7 (1.5)**	84.8 (7.2)	77.0 (4.0)
	0.31 (0.07)	0.30 (0.08)	0.27 (0.04)	**0.25 (0.04)**	0.34 (0.15)	0.51 (0.14)
R Amygdala	79.9 (18.7)	86.3 (4.5)	87.9 (2.7)	**88.6 (2.7)**	85.8 (5.2)	77.5 (2.1)
	2.35 (8.89)	0.30 (0.11)	0.26 (0.06)	**0.25 (0.06)**	0.32 (0.14)	0.48 (0.07)
All	90.3 (7.1)	91.6 (3.6)	92.1 (2.8)	**92.4 (2.5)**	91.7 (4.7)	83.9 (4.5)
	0.41 (2.64)	0.22 (0.07)	0.21 (0.05)	**0.20 (0.04)**	0.24 (0.09)	0.42 (0.09)

Table 1: Reliability in the OASIS scan-rescan dataset using the raw unfiltered manual labellings. Each table cell reports the mean Dice coefficient (standard deviation) as a percentage on top and the mean MHD (standard deviation), in millimeters, on bottom. The top performing method is emboldened in each row.

	CNN-B	CNN-SP	CNN-SP-D	CNN-SP-D + DA	FIRST
L Caudate	84.3 (3.3)	84.3 (5.0)	85.5 (3.1)	**85.7 (2.3)**	72.2 (6.3)
	0.45 (0.16)	0.48 (0.37)	0.38 (0.10)	**0.37 (0.07)**	0.82 (0.20)
R Caudate	84.9 (3.4)	85.8 (2.7)	86.0 (2.5)	**86.2 (2.4)**	69.0 (7.2)
	0.42 (0.17)	0.37 (0.11)	0.36 (0.08)	**0.35 (0.06)**	0.88 (0.22)
L Putamen	86.4 (2.1)	87.3 (2.5)	87.6 (2.3)	**87.8 (2.6)**	84.0 (2.1)
	0.44 (0.08)	0.41 (0.09)	0.39 (0.09)	**0.39 (0.09)**	0.58 (0.08)
R Putamen	87.3 (1.9)	87.7 (1.7)	87.7 (2.0)	**88.0 (1.6)**	83.7 (2.5)
	0.42 (0.07)	0.39 (0.06)	0.41 (0.08)	**0.39 (0.06)**	0.62 (0.09)
L Thalamus	88.7 (1.4)	88.7 (2.8)	88.7 (3.8)	**89.5 (1.6)**	85.6 (2.1)
	0.54 (0.08)	0.60 (0.35)	0.57 (0.23)	**0.53 (0.09)**	0.84 (0.11)
R Thalamus	89.9 (0.9)	89.9 (1.2)	90.2 (1.5)	**90.7 (0.9)**	87.1 (1.3)
	0.49 (0.05)	0.51 (0.08)	0.50 (0.09)	**0.48 (0.05)**	0.76 (0.07)
L Hippocampus	81.2 (3.8)	82.1 (4.7)	83.9 (1.9)	**84.7 (1.6)**	79.6 (1.6)
	0.50 (0.14)	0.60 (0.59)	0.43 (0.08)	**0.42 (0.14)**	0.55 (0.07)
R Hippocampus	80.4 (4.2)	82.3 (3.8)	83.0 (2.1)	**84.5 (1.3)**	79.5 (1.9)
	0.54 (0.21)	0.56 (0.49)	0.46 (0.09)	**0.40 (0.06)**	0.55 (0.06)
L Pallidum	80.2 (4.2)	82.4 (3.8)	82.6 (3.6)	**83.6 (4.7)**	76.1 (6.3)
	0.55 (0.12)	0.51 (0.13)	0.50 (0.11)	**0.47 (0.13)**	0.74 (0.26)
R Pallidum	81.4 (3.8)	83.3 (3.4)	82.9 (4.6)	**83.9 (3.1)**	75.2 (6.2)
	0.51 (0.10)	0.46 (0.10)	0.48 (0.14)	**0.44 (0.09)**	0.77 (0.22)
L Amygdala	69.6 (16.9)	75.3 (4.1)	76.1 (3.9)	**77.8 (3.7)**	69.2 (9.0)
	2.50 (8.31)	0.58 (0.11)	0.59 (0.15)	**0.54 (0.11)**	0.86 (0.38)
R Amygdala	74.5 (4.6)	75.8 (5.1)	77.5 (2.6)	**79.0 (3.3)**	71.0 (4.7)
	0.57 (0.16)	0.58 (0.17)	0.52 (0.07)	**0.50 (0.11)**	0.74 (0.13)
All	82.4 (8.1)	83.8 (5.7)	84.3 (5.1)	**85.1 (4.5)**	77.7 (8.0)
	0.66 (2.47)	0.50 (0.29)	0.46 (0.14)	**0.44 (0.11)**	0.73 (0.22)

Table 2: Accuracy in the OASIS scan-rescan dataset using the raw unfiltered manual labellings. Each table cell reports the mean Dice coefficient (standard deviation) as a percentage on top and the mean MHD (standard deviation), in millimeters, on bottom. The top performing method is emboldened in each row.
